# Metabolomic profile and its association with the diagnosis of prostate cancer: a systematic review

**DOI:** 10.1007/s00432-024-06058-w

**Published:** 2024-12-31

**Authors:** Angela Patricia Salinas Pita, Mildrey Mosquera Escudero, Eliecer Jiménez-Charris, Herney Andrés García-Perdomo

**Affiliations:** 1 Program in Biomedical Sciences, Universidad del Valle, Cali, Colombia; 2 Department of Physiological Sciences, Basic Science School, Nutrition Group, Universidad del Valle, Cali, Colombia; 3 Division of Urology/Urooncology, Department of Surgery, School of Medicine, Universidad del Valle, Calle 4 B # 36-00, Cali, Colombia

**Keywords:** Metabolites, Metabolomics, Prostate cancer

## Abstract

**Objective:**

To determine the association of a metabolomic profile with the diagnosis of localized prostate cancer.

**Methods:**

We conducted a search strategy in MEDLINE (OVID), EMBASE, LILACS, and the Cochrane Central Register of Controlled Trials (CENTRAL) from 2008 to the present. We included Clinical trials and analytical and descriptive observational studies that reported metabolite results and metabolite profiles in serum, tissue, urine, and seminal fluid. All studies used metabolomic techniques such as MS and MRI to identify patients with localized prostate cancer compared with patients without cancer. We used QUADAS 2 to assess the risk of bias.

**Results:**

We found 1248 studies with the search strategy. Finally, 14 case–control studies were included. Serum was the primary sample to identify the metabolites. Low concern was found regarding applying the index test and the reference standard in assessing the risk of bias. The metabolites of interest associated with establishing a metabolomic profile in the diagnosis of localized prostate cancer were amino acids, lipids, androgens, estrogens, nucleotides, and histidine metabolism.

**Conclusion:**

Disturbances in the metabolism of fatty acids, amino acids, nucleotides, and steroid hormones were identified, suggesting the presence of localized prostate cancer. Importantly, serum samples showed an increase in amino acid levels. Glutamate and aspartic acid stand out among the amino acids that register high levels. In addition, glycine and serine were consistently decreased metabolites in the three kinds of biological samples analyzed.

**Supplementary Information:**

The online version contains supplementary material available at 10.1007/s00432-024-06058-w.

## Introduction

Prostate cancer (PCa) is one of the most frequent neoplasms in the male population worldwide (Kachur 2018). It is estimated that one in seven men will be diagnosed with PCa, and in the long term, one in 38 men will die from the disease. In Colombia, mortality from this disease amounts to 3.4% of cases per year (Luis et al. 2014). The incidence rate of this malignant pathology will increase if the approach is not improved. Raising early detection rates and applying measures that have a positive impact on patient outcomes, that is, reducing advanced cases of the disease, will also lower the costs incurred by public health systems (Esquivel Parra et al. 2017).

Timely diagnosis of PCa is essential to make complications and deaths less likely. At present, prostate-specific antigen together with digital rectal examination are the methods mainly used in the clinic to detect it (Esquivel Parra et al. 2017). However, these have poor diagnostic performance and are susceptible to false-positive and false-negative results. Other methods have been studied for both prostate hyperplasia and prostate cancer, but their validity in the general population is still limited (Draisma et al. 2009; Bickers and Aukim-Hastie 2009). This is why there is great interest in investigating new strategies that will lead to better ways to diagnose this sensitive pathology.

Metabolomics is a powerful phenotypic analysis tool used to understand health and disease processes. It provides information on the biology of a living being and the changes that occur in various metabolic processes according to their interaction with environmental factors and genetic determinants. PCa is characterized by modifications in the cellular microenvironment and transformation of cells. As a result of these changes, intra- and extracellular regulatory mechanisms are affected, giving rise to mutant cells. These transformed cells have perturbed processes that are reflected in the way they obtain nutrients, their increased energy demands, and their other adaptations for purposes such as survival (Dang et al. 2019). As we have come to understand such modifications in different disease states, molecules that are products and intermediates of cellular metabolism have attracted great interest, and the discipline that studies all these molecules is called metabolomics.

In recent years, the growing interest in omics sciences, of which metabolomics is a part along with genomics, transcriptomics, and proteomics, has focused on the understanding of a biological system in an integrated way. Now many researchers aim to establish a profile of metabolites for this biological system (cells, tissues, and organism) that reflects the general physiological state and allows the measurement and identification of molecules associated with a disease process (Dinges et al. 2019; Rochfort 2005). A diverse set of methods and tools are available for the study of cancer, and its focus has been broad, due to the multiplicity of molecular signaling pathways involved that provide negative or positive regulatory signals in the control of cell proliferation, a process that is altered in cancer cells (Rubakhin et al. 2011; Gonzalgo et al. 2016; Hanahan and Weinberg 2011). The cellular modifications in cancer cells generate different levels of metabolites than normal cells do, which could be targeted by a diagnostic tool; in this way, the body’s metabolomic profile could reflect direct malignant transformation relationships in the prostate tissue, which could be detected in different fluids of the body. We aimed to determine the association of the metabolomic profile with the diagnosis of localized prostate cancer in diverse samples.

## Methods

We conducted this systematic review according to Cochrane recommendations and following the PRISMA Statement.

### Eligibility criteria

#### Study designs

We included clinical trials, analytical observational, and cross-sectional studies. In addition, molecular studies were conducted in humans.

### Participants

We included studies identifying metabolites and metabolomic profiles in serum, urine, tissue, and seminal fluid in which metabolomic techniques, such as mass spectrometry (MS) and magnetic resonance imaging (MRI), were applied to identify patients with localized prostate cancer.

#### Primary outcome

Establishing the association between metabolites and/or metabolomic profiles with the diagnosis of prostate cancer in comparison with the control patients.

#### Exclusion criteria

Patients who had received antibiotic treatment with steroids or treatment for prostate cancer, other pre-existing neoplasm, urological pathologies, or infections that led to the use of antibiotics.

### Information sources

Sources such as MEDLINE (OVID), EMBASE, LILACS, and the Cochrane Central Register of Controlled Trials (CENTRAL) were searched from 2008 to the present (Appendix 1). To saturate the literature, we also searched for references of relevant articles material obtained from conferences, databases, thesis works, Open Grey, Google Scholar, and clinicaltrials.gov. In cases where it was necessary to obtain additional information, the authors were contacted by email. There were no language or time restrictions.

### Data collection

Two investigators reviewed each reference by title and abstract. A full-text search of relevant studies was performed, predetermined inclusion and exclusion criteria were applied, and data was extracted. Disagreements were resolved by consensus, and a third party clarified the conflict when the disagreement could not be resolved.

Two trained people used a standardized form and independently obtained information from each article: study design, geographic location, author names, title, objectives, inclusion and exclusion criteria, number of patients included, censoring during follow-up, time, definitions of outcomes, outcomes and partnership measures, and source of funding.

### Risk of bias

We assessed the risk of bias with the QUADAS 2 tool, and RevMan 5.4 was used for graphical representation.

### Data analysis/synthesis of results

No quantitative synthesis was performed, given the heterogeneity of the information found.

## Results

### Study selection

**Fig. 1:**
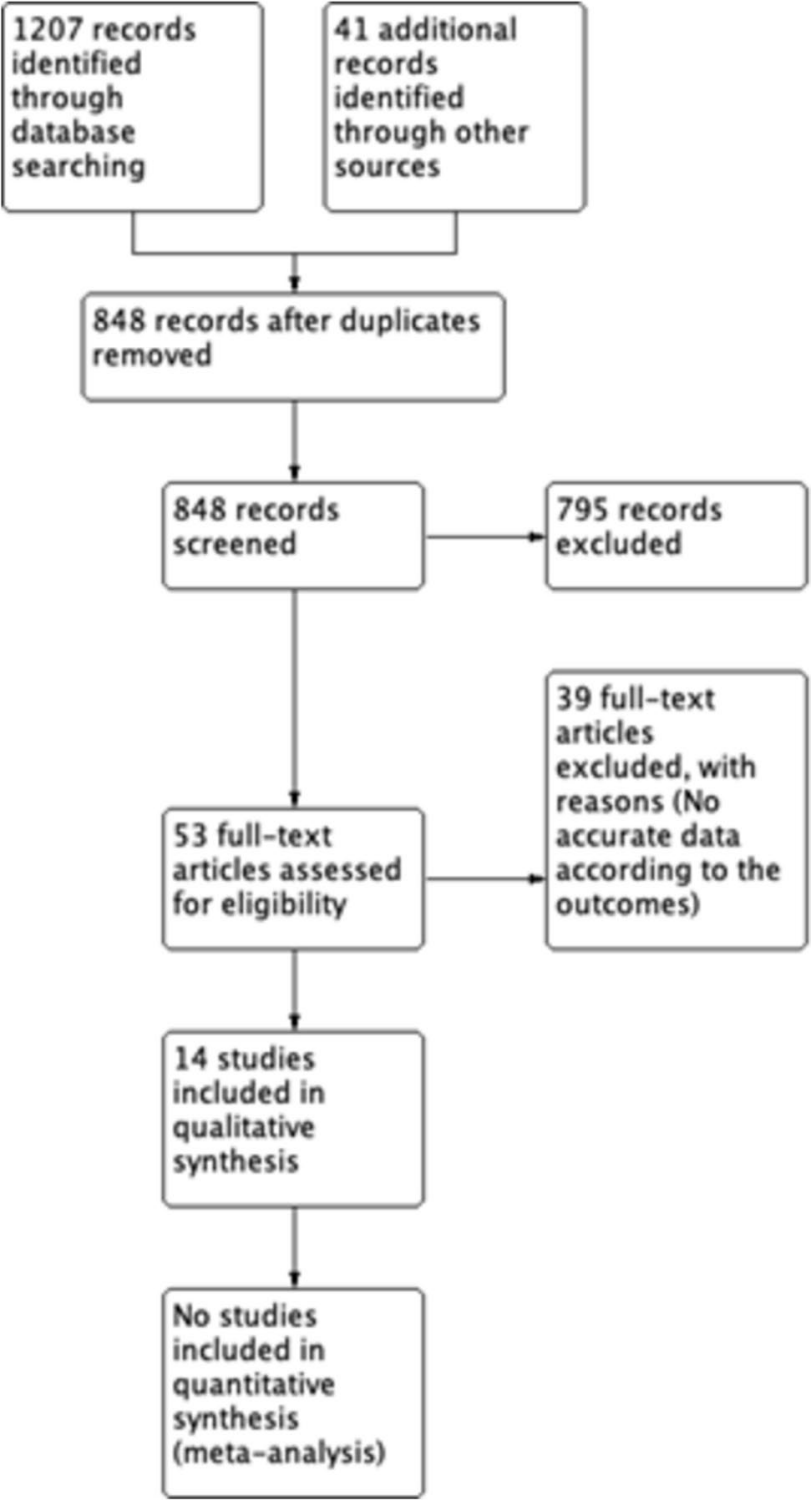
Flowchart of study inclusion

A total of 1248 studies were identified with the search strategy. After reviewing the title, abstract, and full text and after eliminating duplicate articles, 14 studies were included for qualitative analysis (Huan et al. 2016; Osl et al. 2008; Kosti et al. 2011; Giskeødegård et al. 2015; Struck-Lewicka et al. 2015; Zang et al. 2014; Vogel et al. 2014; Koutros et al. 2013; Barocas et al. 2011; Albanes et al. 2016; Patel et al. 2014; Butler et al. 2021; Falegan et al. 2021; Cao et al. 2011) (Fig. 1).

### Characteristics of the included studies

The studies were published between 2008 and 2021. All included studies were case control. Eight were done in North America (Huan et al. 2016; Kosti et al. 2011; Zang et al. 2014; Koutros et al. 2013; Barocas et al. 2011; Albanes et al. 2016; Patel et al. 2014; Falegan et al. 2021), four in Europe (Osl et al. 2008; Giskeødegård et al. 2015; Struck-Lewicka et al. 2015; Vogel et al. 2014), one in Asia (Cao et al. 2011), and one in Oceania (Butler et al. 2021). A total of 10,189 men were evaluated: 5025 individuals with prostate cancer and 4895 controls (Fig. 1). One of the studies included a female group (20 women) for comparison of metabolites in urine (Patel et al. 2014). The age range of the participants was 45–85 years. One study included a group of younger participants in both study groups (average age 49 years) (Vogel et al. 2014). The participants with cancer had an age range of 54–65 years, and the controls had an age range of 49–65 years. One of the studies divided the population into a validation and training cohort (Koutros et al. 2013), without statistically significant differences between the compared groups, and used healthy prostate tissue adjacent to PCa tissue as the positive-control tissue (Table 1).

**Table 1:** Characteristics of the included studies

Study	Country	Sample type	Analytical platform	Study population (n)	Subpopulation	Other population involved	Healthy control population							
							N	Age (mean ± SD)	PSA (average, range)	BMI kg/m^2^ (average ± range)	Smoker (n of individuals:%)	History of PCa (n of individuals;%)	Biopsy Study	Tumor Score Gleason (average, range)
Huan et al. (2016)	Canada	Fabric	LC MS	46	Subdivided into discovery and validation cohort 1 and 2	Only population with PCa and adjacent tissue (without cancer) take controls	NR	NR	NR	NR	NR	NR	NR	NR
Osl et al. (2008)	Austria	Serum	LC—MS	319	3 groups: (1) 120 in group GS 6; (2) 114 in control; (3) 85 the 8–10 group	No	114	NR	NR	NR	NR	NR	NR	NA
Kosti et al. (2011)	USA	Urine	LC/MS–MS	154	NA	77 controls, 37 negative biopsy, remaining healthy patients	77	63.40 (6.81)	< 4 ng/ml: 70(92%); > 4 ng/ml: 6 (8%)	25.28 (3.05)	28 (37)	23 (35)	37 negative biopsy	NA
Giskeødegård et al. (2015)	UK	Serum and Plasma	LC–MS/MS; GC‒MS	50	NA	No	21	62.6 (52–69)	1.2 (0.3–2.6)	26.2 (21.0–40.4)	NR	NR	NR	NA
Struck-Lewicka et al. (2015)	Poland	Urine	HPLC-TOF/MS, GC-QqQ/MS (LC–MS GC‒MS)	64	NA	No	32	52.9 ± 10.7	NR	27.0 ± 5.1	NR	NR	NR	NA
Zang et al. (2014)	USA	Serum	UPLC-MS/MS	114	NA	NA	50	NR	NR	NR	NR	NR	NR	NR
de Vogel et al. (2014)	Norway	Serum	LC–MS/GC‒MS	6000	NA	NA	3000	49.1 (8.7)	NR	25.0 – < 30.0	1159 (39.7%)	NR	NR	NA
Koutros et al. (2013)	USA	Serum	LC—MS	2234	NA	NA	1112	68.1 (5.5)	NR	25–29	122 (11%)	97 (8,7%)	Yes	NA
Cao et al. (2011)	China	Urine	LC MS	110	SI: (1) individuals with PSA < 20 ng/ml—(2): all patients	20 between women and men to quantify sarcosine	39	50–79 (67.68 ± 1.10)	Rango 1.27–19.27 (7.40 ± 0.60)	ND			YES NEM	NA
Albanes et al. (2017)	USA	Serum	LC/MS—GC/MS	337	Patients with localized disease were selected: T2 (71) and separated from other populations T3 (51); T4 (15)	NA	200	59.3	1.4	26	19.4	3.5%	Yes	NA
Patel et al. (2014)	USA	Serum	(ESI–MS/MS)	133	NA	NA	76	50–60	ND	ND	ND	ND	ND	ND
Butler et al. (2021)	Belgium in collaboration with Australia	Fabric	MALDI/ESI–MS/MS	111	To controls; No treatment; B treatment with enzalutamide	No	21	ND	ND	ND	ND	ND	ND	ND
Falegan et al. (2021)	Canada	Seminal fluid	NMR	64	No	No	13	NR	6.1 ± 3	NR	NR	NR	NR	NA
Barocas et al. (2011)	USA	Urine	GC—MS	500	Case, high-grade intraepithelial neoplasia, Control	140 patients with high-grade intraepithelial neoplasia	160	67 ± 7.7	< 4 ng/ml: 51(32.3%);entre 4 y 10 ng/ml: 91 (57.6%); > 10 ng/dl: 16 (10.1%)	< 25: 20(12.5%);entre 25–30 ng/ml: 88 (55%); > 30: 52 (32.5%)	11 (9.8%)	29 (25.4%)	Yes	NA

Study	Prostate volume (average, range)	Case population (CaP)								
		N	PSA (average, range)	Age (mean ± SD)	Biopsy Study	Tumor Score Gleason (average, range)	BMI (average, range)	Smoker (n of individuals;%)	History of PCa (n of individuals;%)	Prostate volume (average, range)
Huan et al. (2016)	NR	46	8.39 (0.73–49.4)	59.3 (49.9–70.4)	Yes	SG 3 + 3, 3 + 4, 4 + 3, 4 + 4 Rango 3–4	28.1 (33.5–21.8)	NR	NR	NR
Osl et al. (2008)	NR	205	NR	NR	Yes	120 GS 6 individuals; 85 en GS 8–10 (Rango de 6–10)	NR	NR	NR	NR
Kosti et al. (2011)	NR	77	< 4 ng/ml: 19 (25); > 4 ng/ml: 58 (75)	63.71 (8.27)	Yes	SG 6:42 (52%); SG 7:24 (31%); NR 13 (17%) (Rango 6–7)	24.39 (2.59)	2 (3)	15 (20)	n/a
Giskeødegård et al. (2015)	48.7 (21–110)	29	11.6 (4.3–50.4)	65.8 (58–76)	Yes	7.0 (Rango 6–9)	26.3 (18–34)	NR	NR	39.6 (19–130)
Struck-Lewicka et al. (2015)	NR	32	NR	64.2 ± 8.1	ND	NR	28.5 ± 4.9	NR	NR	NR
Zang et al. (2014)	NR	64	NR	Rango 49 − 65, (mean 59 ± 4)	SI	Total OS was not achieved, only 3 + 3:13 patients were reported; 3 + 4:27 patients;	NR	NR	NR	NR
de Vogel et al. (2014)	NR	3000	NR	49.1 (8.7)	Yes	NR	25.0 – < 30.0	1142 (39.4%)	NR	NR
Koutros et al. (2013)	NR	1122	NR	68.3 (5.4)	Yes	NR	25–29	78 (7%)	203 (18.1)	NR
Cao et al. (2011)	19.31–55.62 (31.07 ± 0.78)	71	1.67 – 17.50 (7.57 ± 0.44)	53–79 (68.14 ± 0.70)	Yes	reported < 6, 7, > 8	ND	NR	NR	19.31–55.62 (31.07 ± 0.78)
Albanes et al. (2017)	NR	71	8.9	59.8	Yes	NR, provide classification T2, T3, T4	27	18.4	5.3%	NR
Patel et al. (2014)	ND	57	ND	61–70	ND	ND	ND	ND	ND	ND
Butler et al. (2021)	ND	47	ND	ND	ND	ND	ND	ND	ND	ND
Falegan et al. (2021)	NR	51	6.1 ± 3	60 ± 6	SI	NR	NR	NR	NR	NR
Barocas et al. (2011)	46.8 cc	200 but 4 were excluded	< 4 ng/ml:23(11.7%);entre 4 y 10 ng/ml: 122 (61.9%); > 10 ng/dl: 52 (26.4%)	67.5 ± 7	Yes	100 pacientes SG 3 + 3; 100 pacientes SG 4 + 3	< 25: 32(16.2%);entre 25–30 ng/ml: 96 (48.5%); > 30: 70 (35.3%)	18 (13.5)	28 (20.9%)	40 cc

*USA* United States, *UK* United Kingdom, *SD* Standard deviation, *NR* not reported, *NA* not available, *NA* not applicable, *NEM* no evidence of malignancy, *LC–MS* liquid chromatography coupled with mass spectrometry, *GC–MS* gas chromatography coupled to mass spectrometry, *UPLC–MS/MS* high-performance liquid chromatography coupled with tandem mass spectrometry, *MALDI/ESI–MS/MS* desorption by laser ionization assisted by tandem mass spectrometry, *ESI–MS/MS* electrospray ionization coupled to mass spectrometry, *HPLC-TOF/MS* high-quality liquid chromatography coupled with time of flight in mass spectrometry, *NMR* nuclear magnetic resonance

The samples used for metabolomic analysis were heterogeneous: two studies evaluated prostate tissue, seven studies analyze serum, four studies analyze urine samples, and one study use seminal fluid (Table 1). Of these, 13 studies (Huan et al. 2016; Osl et al. 2008; Kosti et al. 2011; Giskeødegård et al. 2015; Struck-Lewicka et al. 2015; Zang et al. 2014; Vogel et al. 2014; Koutros et al. 2013; Barocas et al. 2011; Albanes et al. 2016; Patel et al. 2014; Butler et al. 2021; Cao et al. 2011) performed a metabolomic analysis using an analytical technique coupled with mass spectrometry, followed by identification of the compounds using libraries. Only one study used nuclear magnetic resonance (NMR) for metabolomic analysis (Falegan et al. 2021) (Table 1).

The levels of prostate specific antigen (PSA) were reported by six studies, which measured a range of 1–20 ng/ml for healthy patients and 1–50 ng/ml for the group with PCa (Kosti et al. 2011; Barocas et al. 2011). Eleven of the studies reported biopsy results for PCa patients, which were Gleason scores of 3 + 3 through 4 + 4 (6 through 8) (Huan et al. 2016; Osl et al. 2008; Kosti et al. 2011; Giskeødegård et al. 2015; Zang et al. 2014; Vogel et al. 2014; Koutros et al. 2013; Barocas et al. 2011; Albanes et al. 2016; Falegan et al. 2021; Cao et al. 2011), and three studies did not report the result of the Gleason score (Struck-Lewicka et al. 2015; Patel et al. 2014; Butler et al. 2021; Mueller-Lisse et al. 2001). In the control groups, only five studies reported that these subjects’ biopsies did not present evidence of malignancy in the prostate (Huan et al. 2016; Osl et al. 2008; Giskeødegård et al. 2015; Zang et al. 2014; Vogel et al. 2014) (Table 1).

Supplementary Table 1 describes all the metabolites in serum samples. The metabolites that were reported in more than one study are written in bold. (Supplementary Table 1). The remaining metabolites measured in samples other than serum are listed in Supplementary Table 1.

In the serum samples, a total of 135 metabolites were reported with a predominance of lipid compounds, such as lysophosphatidylcholines, amino acids, compounds of the intermediate metabolism of the Krebs cycle, and some metabolites related to caffeine, such as xanthine’s.

In the prostate tissue samples, 39 metabolites and/or lipids were reported, of which spermidine, uracil, 2,3-diaminopropionic acid + HPO_3_, and lipids as phosphatidylinositol (PI) fractions showed an increase in the samples taken from patients with PCa when comparing them with healthy subjects.

For the urine samples, 85 metabolites were reported. Most corresponded to estrogenic metabolites, but amino acids and purine bases were also found.

Only one study evaluated seminal fluid. It identified seven differential metabolites, one of them an intermediary of the Krebs cycle, pyruvate; three amino acids, lysine, valine, and glycine; a compound derived from purines, xanthine; a neurotransmitter, O-acetylcholine; and a monosaccharide, fructose. Their behavior was heterogeneous. Three metabolites, lysine, glycine, and fructose had decreased expression in the samples from patients with PCa, whereas xanthine, pyruvate, valine, and O-acetylcholine were upregulated in this population (Cao et al. 2011).

In total, 17 metabolites were reported in more than one study (Supplementary Table 3 and Figure 3). Of these molecules, glycine was found to be decreased in three different body fluids (serum, urine, and seminal fluid), while pyruvate, valine, serine, lysine, and azelaic acid were present in two of the fluids studied, where they were consistently up- or downregulated. The other molecules appeared in more than one matrix but with inconsistent behavior. (Supplementary Table 3).

### Differential behavior of metabolites according to the sample analyzed

In Giskeødegård et al. (2015), lipids such as phosphatidylcholines with different carbon chain lengths were decreased in serum samples. This same group of compounds with chain lengths of 40 and 42 carbons was reported in high concentrations in the serum of cancer patients by Patel et al. (2014). In prostate tissue, according to Butler et al. (2021), metabolites with a variety of carbon chain lengths were found at high concentrations. The study by Zang et al. (2014), which was carried out in serum, found lysophosphatidylcholines with different lengths of carbon chains and different numbers of double bonds had differential concentrations in samples from patients with PCa.

Some metabolites, such as sarcosine, had discrepancies in their behavior in serum. Cao et al. (2011) and Koutros et al. (2013) found sarcosine to be upregulated in PCa urine and serum samples, unlike Vogel et al. (2014), who found this metabolite downregulated in the serum of patients with PCa.

Amino acids such as lysine and glycine, among others, were reported in more than one study, showing differential behaviors depending on the type of sample. Sarcosine and pyruvate were reported in more than one study, but with differential behavior between samples, as already described (Supplementary tables 2 and 3).

Phenylalanine behaved differentially in serum samples, presenting high levels in patients with prostate cancer in Giskeødegård et al. (2015) and lower levels in samples of patients with malignancy, according to Zang et al. (2014).

Falegan et al. (2021) reported increased lysine metabolites in seminal fluid samples, and Giskeødegård et al. (2015) reported increased lysine metabolites in serum samples.

Struck-Lewicka et al. (2015) reported that xanthines in association with different chemical groups had decreased concentrations in PCa urine, while Falegan et al. (2021) reported that these metabolites had higher concentrations within seminal fluid samples taken from patients with localized cancer (Albanes et al. 2016).

Lipid molecules such as lysophosphatidylcholine and phosphatidylcholine, with chains of 16 and 18 carbons, were consistently decreased in serum samples, as seen in Giskeødegård et al. (2015), Osl et al. (2008) and Zang et al. (2014).

**Fig. 2:**
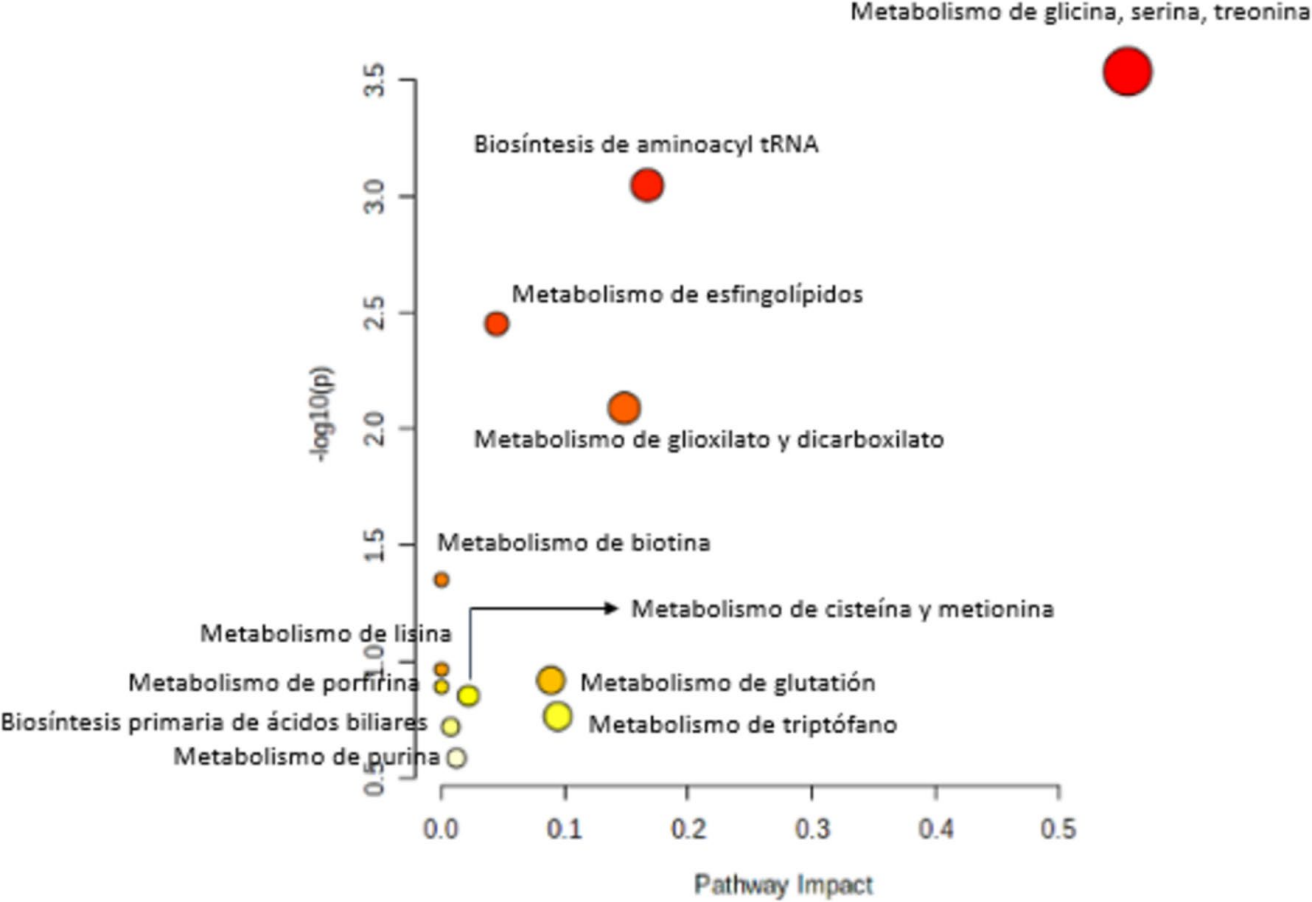
Analysis of metabolic pathways involved in the process of malignancy

Figure 2 presents the enrichment analysis of pathways that we ran on the metabolites that were altered in several studies. These compounds were related to the metabolism of glycine, serine, and threonine; dicarboxylate metabolism; tRNA biosynthesis; cysteine and methionine metabolism; and the citrate cycle.

### Risk-of-bias analysis

Supplementary Figs. 1A and 1B show the results of the risk-of-bias analysis; the risk-of-bias analysis showed positive results; the risk of bias was low in most of the studies, and there were only difficulties in the selection process of the participants within the studies. thanks to the judicious planning of the methods and application of the diagnostic test in the populations under comparison (Huan et al. 2016). Seven studies presented high risk and five presented unclear risk in the selection of patients (Kosti et al. 2011; Giskeødegård et al. 2015; Struck-Lewicka et al. 2015; Zang et al. 2014; Patel et al. 2014; Butler et al. 2021; Falegan et al. 2021) since the description in the papers did not allow a clear interpretation. Almost all the studies had a high risk of bias in the evaluation of the index test. Although the diagnostic test was applied systematically in the PCa patient population, the Gleason score associated with the identification of PCa was not always reported, and it was not applied to all control patients to rule out histological alterations that would modify the patient's condition. Thirteen of the studies were evaluated as having a low risk of bias with respect to the reference standard, patient workflow, and temporality. There was no concern regarding the applicability of patient selection, the index test, or the reference standard (Supplementary Fig. 1A and Fig. 1B).

## Discussion

### Summary of the main findings

**Fig. 3:**
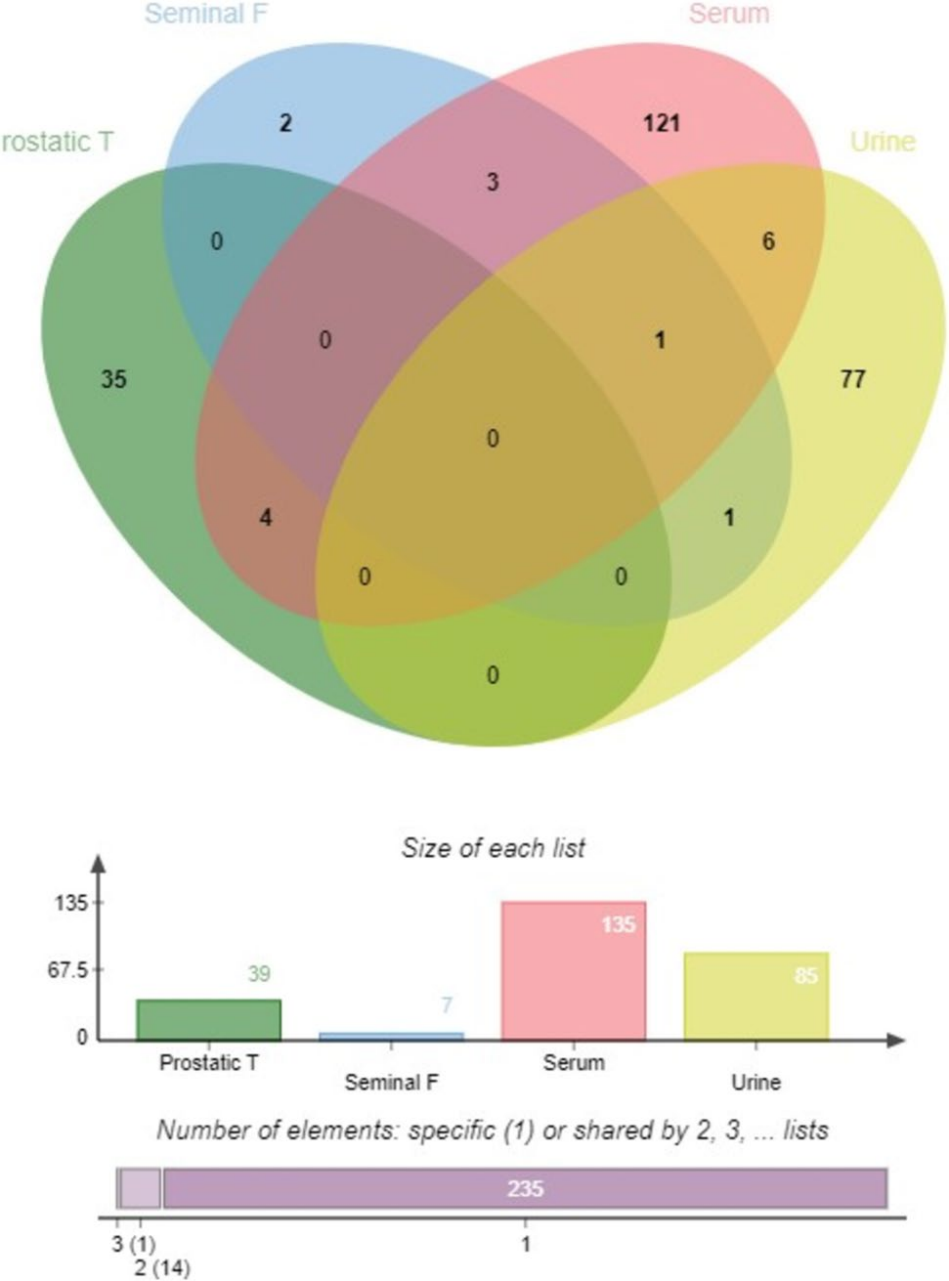
Venn graph summarizing the samples analyzed and the interception of the identified metabolites

In this study, we found more than 260 molecules whose levels in different biological matrices have been associated with PCa. Seventeen molecules were associated with PCa in more than one study. Of these molecules, the amino acid glycine was altered in three different matrices in the same direction. Five molecules were reported in at least two studies, with consistent behavior. Finally, through the analysis of metabolic pathways, we found that the metabolism of both amino acids and sphingolipids is affected during PCa (Fig. 3).

This study also found that several lipid molecules, such as lysophosphatidylcholines and phosphatidylcholines with different lengths in the carbon chain and different numbers of double bonds, were differentially regulated in serum and prostate tissue, with discrepancies in the behavior between analyzed articles. In prostate tissue, a high proportion of monounsaturated lipids of the group of phosphatidylcholines and monounsaturated fatty acids (fatty acyl chains with one double bond) was observed, unlike the findings in the serum, where the lipid types LysoPC and PC were decreased in the samples of patients with prostate cancer.

In this review, it became clear that the body fluid(s) to use for this type of analysis is not unanimous among the scientific community; the most studied was serum, followed by urine. Mass spectrometry has been the most often used analytical technique for this type of study.

### Contrast with the literature

Current techniques for the diagnosis of prostate cancer, such as PSA measurement and digital rectal examination, are not supported by enough evidence to conclude either one alone achieves an unequivocal identification of malignancy in the prostate gland. Metabolomics is a highly dynamic discipline that offers information on the global state of operation of a living system. Metabolomics is the study of a set of molecules, called metabolites, that aims to gather information on dynamic changes at the molecular level (Ivanisevic and Thomas 2018). This valuable tool makes interesting contributions and presents intriguing possibilities for establishing the diagnosis of diseases through an understanding of the molecular mechanisms that underlie pathologies.

### Glucose and lipid metabolism

The malignant transformation of prostate cells requires adaptations in the metabolism of intermediary molecules that meet the high energy requirements of this accelerated cell division process. Taking this postulate into account, glucose and lipid metabolism seem to have a central role (Wu et al. 2014a).

Normal prostate epithelial cells have a low-efficiency energy metabolism during the use of glucose as a substrate for citrate synthesis. In the process of transformation toward malignancy, this metabolism is modified, shifting to a high rate of glycolysis, even under adequate-oxygen conditions, a behavior called the Warburg effect. The particularity of this energy pathway in cells with malignant transformation is independent of the availability of oxygen (Cutruzzolà et al. 2017). Interestingly, glucose consumption is not characteristic of malignant prostate cells in their initial stages; for this reason, positron-emission tomography with ^18^F fluorodeoxyglucose to detect prostate cancer depends on the stage of the disease and the natural history of this prevalent entity in the male population (Jadvar 2016). This finding is related to the finding of glucose at high concentrations in serum samples of patients with localized prostate cancer (Giskeødegård et al. 2015). While glucose provides, although not exclusively, the source of nutrients and energy necessary for the synthesis of macromolecules, lipids are needed for cell proliferation. In this way, malignant prostate cells increase oxygen consumption to sustain overflowing proliferation; consequently, large amounts of citrate that under normal conditions should be secreted behave as intermediaries in the tricarboxylic acid cycle and act as a substrate for de novo synthesis of fatty acids (Wu et al. 2014a). The most representative metabolite changes in the prostate tissue samples were a decrease in the concentration of adenosine monophosphate (AMP) in the samples belonging to patients with prostate cancer, which would seem to reflect the changes in the metabolic activity of ATP and the deactivation of the AMP protein kinase involved in the progression of malignancy, and the increase in the concentrations of uracil and spermidine in the same population (Huan et al. 2016; Mueller-Lisse et al. 2001).

PCa also shows altered regulation of lipids. On the one hand, fatty acid synthesis is unregulated during malignant transformation, becoming constitutively active, and there is no modulation of this pathway by the circulating levels of fatty acids. This situation highlights the activity profile in cancer cells, where lipogenesis is stimulated and, in turn, there is an increase in the expression of lipogenic enzymes resulting from the oncogenic signaling of the PI3K/Akt and HER2 pathways (Liu 2006) In parallel, the literature reveals the dominant role of the bioenergetic pathway of fatty acid oxidation in prostate cancer and the overexpression of enzymes that participate in this process, such as α-methylacyl-CoA racemase, for branched-chain fatty acid transformation (Wu et al. 2014b; Poulose et al. 2018).

In particular, lipid levels showed significant variations among patients with localized T2 prostate cancer, with reduced levels of the glycerophospholipids steryl-arachidonyl-GPE and steryl-linoleyl-GPE, while for cancers that had spread extraprostatically (T3–T4), an increase in the levels of metabolites was evidenced by the steryl-sphingolipids euricoyl-sphingomyelin and myristoyl-sphingomyelin and a decrease in the glycerophospholipids oleoyl-linoleoyl-glycerophosphoinositol (GPI) and palmitoyl-linoleoyl-GPI (Giskeødegård et al. 2015).

### Amino acid metabolism

A wide spectrum of alterations is associated with the metabolism and biosynthesis of amino acids in cells with malignant transformation, and this ability is evident when the cells need to survive unfavorable conditions such as hypoxia, oxidative stress, and increasing energy demands (Strmiska et al. 2019). Several amino acid-like metabolites had altered concentrations in samples obtained from prostate cancer patients. In serum, the main amino acids with high concentrations were histidine, arginine, valine, tyrosine, glutamate, and phenylamine, while glycine was downregulated in serum. In the analysis of the urine samples, low concentrations of alanine, tyrosine, tryptophan, and leucine were present (Vogel et al. 2014;; Struck-Lewicka et al. 2015). One article performed NMR on seminal fluid (Falegan et al. 2021), which identified valine as upregulated in samples of patients with localized prostate cancer compared to controls, while the levels of lysine and glycine found in the same patients were much lower than those in healthy patients.

### Choline metabolism

Choline is an essential nutrient, being necessary for the synthesis of acetylcholine and membrane signaling phospholipids (phosphatidylcholine and sphingomyelin) and is a source of methyl groups. Choline metabolism has been associated with malignant transformation characterized by a high rate of proliferation and an increase in phosphocholine and other choline-containing compounds (Awwad et al. 2012). In samples of serum and prostate tissue from patients with prostate cancer, an increase in the levels of metabolites containing choline was observed (Huan et al. 2016). Additionally, in the studies in which samples of prostate tissue were evaluated, it was possible to establish the concentration of polyamines as statistically significant metabolites in cancer patients (Huan et al. 2016; Butler et al. 2021). One of these studies found that a type of polyamine, spermine, was present in high concentrations in these patients (Huan et al. 2016). This polyamine is involved in cell transformation processes and has been associated with tumor progression (Soda 2011). Its increase could be explained by the activity of the enzyme ornithine decarboxylase followed by the reduction in its secretion, through which it would reach high levels in cancer patients (Shantz and Levin 2007).

To make a timely diagnosis of prostate cancer, molecules of interest have been measured, such as metabolites whose analysis in the different body fluids can indicate the state of health or disease. Establishing standard ways to obtain these molecules will allow greater comparison of different study subjects. Blood is an easily, minimally invasively obtained biological fluid on which chemical analyses have been carried out for more than 70 years, and there are reference tables for different molecules, such as ions, gases, and more than 100 metabolites (Psychogios et al. 2011; Grant and Butt 1970). Urine is also easily obtained noninvasively and can offer information on the interaction between the individual and their surrounding internal environment (Zhou et al. 2017). The study of the metabolites present in the urine is ideal because of the communication between the lumens of tissues and the urinary space. In addition, urinalysis is valuable because what appears in this fluid derives from the production, use, and renal filtration of molecules (Kim et al. 2011). Few articles have analyzed the metabolites present in seminal fluid. Not unlike urine, seminal fluid is an adequate option to study the possible alterations in prostate tissue because it is obtained in a relatively minimally invasive way.

### Clinical effects of the application of metabolomics in PCa

Metabolomics has emerged as a promising tool in the field of prostate cancer, as it allows a comprehensive analysis of metabolites present in biological fluids such as urine, blood or prostate tissue. This technology facilitates the identification of specific biomarkers that can improve early diagnosis, assessment of disease progression and response to treatment. In the context of prostate cancer, metabolomics can reveal alterations in key metabolic pathways, such as lipid, energy and amino acid metabolism, that are involved in carcinogenesis and therapy resistance. These advances not only enhance the accuracy of detection, but also open up new perspectives in personalized medicine, enabling a more tailored and effective approach to prostate cancer management.

This systematic review proposes an updated analysis of the information identifying potential biomarkers that can be evaluated in particular situations and allow staging of patients, predicting the behavior of the pathology and its response to treatment, becoming an important tool for diagnostic support.

### Strengths and limitations

One of the most notable findings of this study is the identification of certain molecules, such as glycine, lysine, and membrane lipids, which show a consistent association with prostate cancer (PCa) across different studies and in different biological fluids. These molecules are important because their presence or alterations in levels may be related to the biology of prostate cancer. This consistency across various studies suggests that these molecules might not be mere accidental markers but could have a relevant biological function in the development process of the disease. Furthermore, as these molecules have been identified in different body fluids, such as blood, urine, and plasma, this increases the robustness of their association with PCa, as it indicates that these biomarkers are determinable in various clinical contexts, making them candidates to be used in diagnostic and monitoring tests. Another strong point of this study is the detailed analysis of the metabolic pathways involved in prostate cancer. By analyzing metabolic alterations, researchers can identify underlying biological mechanisms that could be involved in cancer. Metabolic pathways are sets of biochemical reactions that occur in cells, and alterations in these pathways are a hallmark of many diseases, including cancer. The researchers in this study have managed to identify specific changes in metabolic pathways in PCa, which may help to understand how cancerous prostate cells develop and spread. This approach provides deeper insight into how tumor cells alter their metabolism to promote their growth, survival, and dissemination. Key molecules involved in these pathways, which are consistently identified across studies, could serve not only as disease indicators, but also as potential therapeutic targets.

Since the observed metabolic alterations can be correlated with disease progression, the identified biomarkers, such as glycine, lysine, and membrane lipids, can be evaluated for use in early detection, treatment monitoring, or prediction of prostate cancer progression.

This identification opens the door to potential advances in diagnosis, such as the creation of noninvasive tests that measure these biomarkers in blood, urine, or other body fluids, which would allow for early detection and continuous monitoring of the disease status.

One of the main limitations of the study is the heterogeneity observed between the studies included in the review. This heterogeneity refers to the substantial differences between the studies in terms of several key variables.

Variables studied: The studies included in the review considered different biomarkers and metabolic factors. This can lead to difficulties in comparing the results directly, since each study could have focused its analysis on a different set of biomarkers or metabolic pathways.

The sample sizes in the studies varied significantly. Some studies included few participants, which may reduce the statistical power of the results, while other studies had large cohorts, which could have influenced the ability to generalize the findings. The follow-up time of the patients was also variable between the studies. Some studies may have followed patients for a short period of time, limiting the ability to identify biomarkers associated with long-term disease progression or recurrence. The included studies used different analytical techniques, which may have led to differences in results. Differences in sensitivity, specificity, and methodology of the approaches used (such as mass spectrometry, liquid chromatography, etc.) could have affected the accuracy of the results and the ability to replicate them in other settings.

Due to methodological variability across studies, researchers encountered difficulties when attempting to synthesize the information. The lack of standardization in the approaches and techniques used in each study may make the overall interpretation of the data difficult and may have reduced the strength of the study.

## Conclusions

Alterations in the metabolism of fatty acids, spermidines, intermediate metabolites of the Krebs cycle, xanthines, and amino acids such as serine and lysine suggest the presence of localized PCa. Importantly, serum samples of PCa patients showed a decrease in lysophosphatidylcholine levels. Among the amino acids, serine and lysine stand out as dysregulated amino acids in the samples of prostate cancer patients, and a derivative of the amino acids, sarcosine, showed discrepancies in its reported up- or downregulation.

Metabolomics will have an important role in diagnosing prostate cancer, but more studies are required to validate and establish a group of metabolites with applicability in the early diagnosis of the disease, to allow appropriate actions to be taken to treat this disease.

## Supplementary Information

Below is the link to the electronic supplementary material.

Supplementary file1 (PNG 72 KB)

Supplementary file2 (PNG 18 KB)

Supplementary file3 (DOCX 30 KB)

Supplementary file4 (DOCX 50 KB)

Supplementary file5 (DOCX 16 KB)

Supplementary file6 (PDF 33 KB)

## Data Availability

No datasets were generated or analysed during the current study.
