# Supplementary figures and images for: Metabolomic profile and its association with the diagnosis of prostate cancer: a systematic review
Source: J Cancer Res Clin Oncol. 2024 Dec 31;151(1):29. doi: 10.1007/s00432-024-06058-w (PMC11688254; doi:10.1007/s00432-024-06058-w)

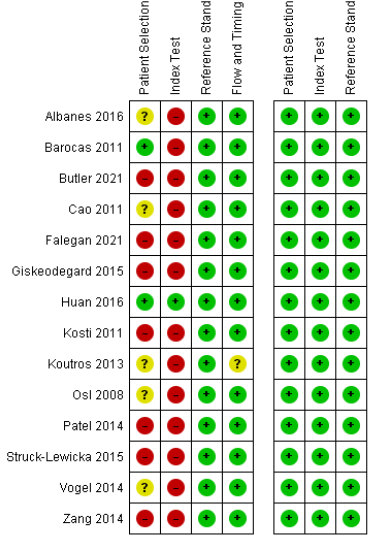

Supplement: Supplementary file 1 — Supplementary file1 (PNG 72 KB) [file 432_2024_6058_MOESM1_ESM.png]

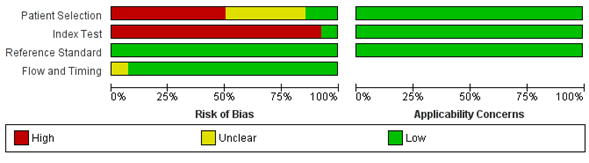

Supplement: Supplementary file 2 — Supplementary file2 (PNG 18 KB) [file 432_2024_6058_MOESM2_ESM.png]
