# Supplementary material for: Metabolomic profile and its association with the diagnosis of prostate cancer: a systematic review
Source: J Cancer Res Clin Oncol. 2024 Dec 31;151(1):29. doi: 10.1007/s00432-024-06058-w (PMC11688254; doi:10.1007/s00432-024-06058-w)
Supplement: Supplementary file 3 — Supplementary file3 (DOCX 30 KB) [file 432_2024_6058_MOESM3_ESM.docx]

| ***Metabolite*** | | ***Authors*** | | ***Fold change or* β** | | ***p value*** | |  |
| --- | --- | --- | --- | --- | --- | --- | --- | --- |
| **Prostate tissue** | | | | | | | |  |
| Spermidine | | Tao Huan et al. (2016) | | (↑) | | < 8.5E-03 | |  |
| Uracil | |  |  | (↑) | | < 8.0E-03 | |  |
| Adenosine monophosphate (AMP) | |  |  | (↓) | | < 4.6E-03 | |  |
| Ophthalmic acid + HPO_3_ | |  |  | (↑) | | < 3.1E-03 | |  |
| 2,3-Diaminopropionic acid + HPO_3_ | |  |  | (↑) | | < 1.7E-02 | |  |
| PI 38:6 | | Butler et al. (2021) | | (↑) | | 6.52E-19 | |  |
| PI 40:6 | |  |  | (↑) | | 1.78E-14 | |  |
| PS 40:8 | |  |  | NR | | 6.40E-14 | |  |
| PC 36:4 | |  |  | (↑) | | 3.51E-11 | |  |
| PC 34:1 | |  |  | (↑) | | 5.81E-10 | |  |
| **PC** 40:4 | |  |  | (↑) | | 1.18E-06 | |  |
| PI 38:5 | |  |  | (↑) | | 4.51E-06 | |  |
| PS 42:8 | |  |  | NR | | 2.20E-05 | |  |
| PS 42:2 | |  |  | NR | | 4.08E-05 | |  |
| PS 42:4 | |  |  | NR | | 8.25E-05 | |  |
| PE 42:6 | |  |  | (↑) | | 0.000149 | |  |
| PI 36:4 | |  |  | (↑) | | 0.000203 | |  |
| PE 42:5 | |  |  | (↑) | | 0.000255 | |  |
| PS 38:4 | |  |  | NR | | 0.000399 | |  |
| PC 38:4 | |  |  | (↑) | | 0.000693 | |  |
| PS 36:2 | |  |  | NR | | 0.000753 | |  |
| PS 36:1 | |  |  | NR | | 0.000789 | |  |
| PC 32:0 | |  |  | (↑) | | 0.000832 | |  |
| PS 38:3 | |  |  | NR | | 0.000942 | |  |
| PC 40:6 | |  |  | (↑) | | 0.001045 | |  |
| PS 38:6 | |  |  | NR | | 0.001116 | |  |
| PE 40:4 | |  |  | (↑) | | 0.001414 | |  |
| PC 38:5 | |  |  | (↑) | | 0.001489 | |  |
| PE 38:7 | |  |  | (↑) | | 0.001681 | |  |
| PI 38:4 | |  |  | (↑) | | 0.001944 | |  |
| PC 36:5 | |  |  | (↑) | | 0.002105 | |  |
| PS 44:6 | |  |  | NR | | 0.002306 | |  |
| PI 36:1 | |  |  | (↑) | | 0.00274 | |  |
| PS 42:9 | |  |  | NR | | 0.003707 | |  |
| PS 40:4 | |  |  | NR | | 0.003719 | |  |
| PE 42:9 | |  |  | (↑) | | 0.005855 | |  |
| PE 40:6 | |  |  | (↑) | | 0.006456 | |  |
| PE 40:8 | |  |  | (↑) | | 0.006487 | |  |
| PC 40:5 | |  |  | (↑) | | 0.008872 | |  |
| **Seminal fluid** | | | | | | | |  |
| **Lysine** | | Falegan et al. (2020) | | (↓) | | < 0.05 | |  |
| **Xanthine** | |  |  | (↑) | | < 0.05 | |  |
| **Pyruvate** | |  |  | (↑) | | < 0.05 | |  |
| **Wisteria** | |  |  | (↓) | | < 0.05 | |  |
| Fructose | |  |  | (↓) | | < 0.05 | |  |
| **Valine** | |  |  | (↑) | | < 0.05 | |  |
| Or acetylcholine | |  |  | (↑) | | < 0.05 | |  |
| **Urine** | | | | | | | |  |
| F2-Isoprostane | | Barocas et al. (2011) | | (↑) | | 0.001 | |  |
| **Sarcosine** | | Cao et al. (2011) | | (↑) | | 0.05 | |  |
| 17-Epiestriol | | Kosti et al. (2011) | | (↓) | | 0.05 | |  |
| 16-Cetoestradiol | |  |  | (↓) | | 0.03 | |  |
| Hydroxybutanoic acid | | Struck-Lewicka et al. (2015) | | (↓) | | 0.035 | |  |
| Cresol | |  |  | (↓) | | 2.4 E -06 | |  |
| Succinic acid | |  |  | (↓) | | 0.011 | |  |
| Benzoate | |  |  | (↓) | | 0.001 | |  |
| Oxoproline | |  |  | (↓) | | 0.001 | |  |
| Leucine | |  |  | (↓) | | 0.005 | |  |
| Indoxil | |  |  | (↓) | | 0.0001 | |  |
| Phenylacetamide | |  |  | (↓) | | 0.0007 | |  |
| Threonic acid | |  |  | (↓) | | 0.002 | |  |
| **Glutamine** | |  |  | (↓) | | 0.028 | |  |
| Ureidoisobutyrate | |  |  | (↓) | | 0.0008 | |  |
| Hydroxyglutarate | |  |  | (↓) | | 6.3 E -09 | |  |
| α-Oxo-benzeneacetic acid | |  |  | (↓) | | 0.029 | |  |
| **xanthine** | |  |  | (↓) | | 0.0004 | |  |
| Acetamidopentanoate | |  |  | (↓) | | 0.005 | |  |
| Dihydroxyquinoline | |  |  | (↓) | | 0.002 | |  |
| Dehydrodeoxy fuconoate | |  |  | (↓) | | 3.2 E -06 | |  |
| Xylonate | |  |  | (↓) | | 0.001 | |  |
| Methylxanthine | |  |  | (↓) | | 0.005 | |  |
| Indole acetate | |  |  | (↓) | | 0.006 | |  |
| Citrulline | |  |  | (↓) | | 8,1501 E -06 | |  |
| Propylmalate hippuric acid | |  |  | (↓) | | 0.049 | |  |
| Hexose | |  |  | (↓) | | 9.52 E 05 | |  |
| DImethyl xanthine | |  |  | (↓) | | 0.006 | |  |
| Tyrosine | |  |  | (↓) | | 2.14 E -06 | |  |
| Dihydroxyphenylpropanoate | |  |  | (↓) | | 0.006 | |  |
| Adrenaline | |  |  | (↓) | | 2.14 E -05 | |  |
| Azelaic acid | |  |  | (↓) | | 0.002 | |  |
| Kinurenate | |  |  | (↓) | | 9.39 E -05 | |  |
| Dihydroxyhipuric acid | |  |  | (↓) | | 0.023 | |  |
| Dimethyluric acid | |  |  | (↓) | | 0.04 | |  |
| Acetyl amino - aminomethyluracil | |  |  | (↓) | | 0.0018 | |  |
| Tryptophan | |  |  | (↓) | | 0.002 | |  |
| Indolactate | |  |  | (↓) | | 0.0002 | |  |
| Hydroxybutane tricarboxylate | |  |  | (↓) | | 1.18 E -05 | |  |
| Trimethyluric acid | |  |  | (↓) | | 0.0002 | |  |
| Glutamyl amniobutyraldehyde | |  |  | (↓) | | 0.007 | |  |
| Propanoylcarnitine | |  |  | (↓) | | 0.005 | |  |
| Pantothenic acid hydroxy-tryptophan | |  |  | (↓) | | 0.036 | |  |
| Butiril carnitine | |  |  | (↓) | | 0.002 | |  |
| C16 sphingosine | |  |  | (↑) | | 0.033 | |  |
| Methylinosine | |  |  | (↓) | | 0.003 | |  |
| Xanthosine | |  |  | (↓) | | 0.004 | |  |
| Octanoylcarnitine | |  |  | (↓) | | 0.002 | |  |
| Methylguanosine | |  |  | (↓) | | 0.0002 | |  |
| Dimethylheptanoylcarnitine | |  |  | (↓) | | 0.003 | |  |
| Dimethylguanosine | |  |  | (↓) | | 0.047 | |  |
| Hydroxysphingosine | |  |  | (↓) | | 0.002 | |  |
| Phytosphingosine/hydroxysphinganine | |  |  | (↑) | | 0.005 | |  |
| Cyclic 3′,5-AMP | |  |  | (↓) | | 0.001 | |  |
| PA (12:0) | |  |  | (↓) | | 0.05 | |  |
| PG (32:1) | |  |  | (↑) | | 0.007 | |  |
| PG (31:4) | |  |  | (↓) | | 0.015 | |  |
| Alanine | |  |  | (↓) | | 3.156 × 10^-9^ | |  |
| Acetic acid | |  |  | (↓) | | 1.016 × 10^-6^ | |  |
| Arabitol | |  |  | (↓) | | 1.210 × 10^-6^ | |  |
| Threonine | |  |  | (↓) | | 4.061 × 10^-6^ | |  |
| Glyceryl glycoside | |  |  | (↓) | | 5.479 × 10^-6^ | |  |
| Sucrose | |  |  | (↓) | | 5.559 × 10^-6^ | |  |
| Propanetricarboxylic acid | |  |  | (↓) | | 5.849 × 10^-5^ | |  |
| Butyric acid | |  |  | (↓) | | 8.094 × 10^-5^ | |  |
| Propionic acid | |  |  | (↓) | | 1.138 × 10^-5^ | |  |
| Propenoic acid | |  |  | (↓) | | 2.166 × 10^-4^ | |  |
| Hydroxyphenylhydroxypropionic acid | |  |  | (↓) | | 4.922 × 10^-4^ | |  |
| Threonic acid | |  |  | (↓) | | 1.161 × 10^-4^ | |  |
| Arabinous | |  |  | (↓) | | 1.727 × 10^-3^ | |  |
| Indole | |  |  | (↓) | | 2.376 × 10^-3^ | |  |
| Hydroxyhippurate | |  |  | (↓) | | 2.388 × 10^-3^ | |  |
| Meso erythritol | |  |  | (↓) | | 3.548 × 10^-3^ | |  |
| Isobutyric acid | |  |  | (↓) | | 4.185 × 10^-3^ | |  |
| 2-Keto-l-gluconic acid | |  |  | (↓) | | 4.192 × 10^-3^ | |  |
| Talosa | |  |  | (↓) | | 5.316 × 10^-3^ | |  |
| Aconitic acid | |  |  | (↓) | | 5.878 × 10^-3^ | |  |
| Sorbose | |  |  | (↓) | | 6.417 × 10^-3^ | |  |
| Isocytric acid | |  |  | (↓) | | 6.613 × 10^-3^ | |  |
| **Serine** | |  |  | (↓) | | 7.866 × 10^-3^ | |  |
| **Wisteria** | |  |  | (↓) | | 1.244 × 10^-3^ | |  |
| Lactose | |  |  | (↓) | | 1.321 × 10^-2^ | |  |
| Hippuric acid | |  |  | (↓) | | 3.187 × 10^-2^ | |  |
| Galactaric acid | |  |  | (↓) | | 3.532 × 10^-2^ | |  |
| Inositol | |  |  | (↓) | | 4.093 × 10^-2^ | |  |
