# Supplementary material for: Metabolomic profile and its association with the diagnosis of prostate cancer: a systematic review
Source: J Cancer Res Clin Oncol. 2024 Dec 31;151(1):29. doi: 10.1007/s00432-024-06058-w (PMC11688254; doi:10.1007/s00432-024-06058-w)
Supplement: Supplementary file 4 — Supplementary file4 (DOCX 50 KB) [file 432_2024_6058_MOESM4_ESM.docx]

|  | | | |  |
| --- | --- | --- | --- | --- |
| ***Metabolite*** | ***Authors*** | ***Fold change or* β** | ***p value*** |  |
| **Prostate tissue** | | | |  |
| Spermidine | Tao Huan et al. (2016) | (↑) | < 8.5E-03 |  |
| Uracil |  | (↑) | < 8.0E-03 |  |
| Adenosine monophosphate (AMP) |  | (↓) | < 4.6E-03 |  |
| Ophthalmic acid + HPO_3_ |  | (↑) | < 3.1E-03 |  |
| 2,3-Diaminopropionic acid + HPO_3_ |  | (↑) | < 1.7E-02 |  |
| PI 38:6 | Butler et al. (2021) | (↑) | 6.52E-19 |  |
| PI 40:6 |  | (↑) | 1.78E-14 |  |
| PS 40:8 |  | NR | 6.40E-14 |  |
| PC 36:4 |  | (↑) | 3.51E-11 |  |
| PC 34:1 |  | (↑) | 5.81E-10 |  |
| **PC** 40:4 |  | (↑) | 1.18E-06 |  |
| PI 38:5 |  | (↑) | 4.51E-06 |  |
| PS 42:8 |  | NR | 2.20E-05 |  |
| PS 42:2 |  | NR | 4.08E-05 |  |
| PS 42:4 |  | NR | 8.25E-05 |  |
| PE 42:6 |  | (↑) | 0.000149 |  |
| PI 36:4 |  | (↑) | 0.000203 |  |
| PE 42:5 |  | (↑) | 0.000255 |  |
| PS 38:4 |  | NR | 0.000399 |  |
| PC 38:4 |  | (↑) | 0.000693 |  |
| PS 36:2 |  | NR | 0.000753 |  |
| PS 36:1 |  | NR | 0.000789 |  |
| PC 32:0 |  | (↑) | 0.000832 |  |
| PS 38:3 |  | NR | 0.000942 |  |
| PC 40:6 |  | (↑) | 0.001045 |  |
| PS 38:6 |  | NR | 0.001116 |  |
| PE 40:4 |  | (↑) | 0.001414 |  |
| PC 38:5 |  | (↑) | 0.001489 |  |
| PE 38:7 |  | (↑) | 0.001681 |  |
| PI 38:4 |  | (↑) | 0.001944 |  |
| PC 36:5 |  | (↑) | 0.002105 |  |
| PS 44:6 |  | NR | 0.002306 |  |
| PI 36:1 |  | (↑) | 0.00274 |  |
| PS 42:9 |  | NR | 0.003707 |  |
| PS 40:4 |  | NR | 0.003719 |  |
| PE 42:9 |  | (↑) | 0.005855 |  |
| PE 40:6 |  | (↑) | 0.006456 |  |
| PE 40:8 |  | (↑) | 0.006487 |  |
| PC 40:5 |  | (↑) | 0.008872 |  |
| **Seminal fluid** | | | |  |
| **Lysine** | Falegan et al. (2020) | (↓) | < 0.05 |  |
| **Xanthine** |  | (↑) | < 0.05 |  |
| **Pyruvate** |  | (↑) | < 0.05 |  |
| **Wisteria** |  | (↓) | < 0.05 |  |
| Fructose |  | (↓) | < 0.05 |  |
| **Valine** |  | (↑) | < 0.05 |  |
| Or acetylcholine |  | (↑) | < 0.05 |  |
| **Serum** | | | |  |
| Ornithine | Osl et al. (2008) | (↓) | < 0.001 |  |
| Serotonin |  | (↓) | < 0.001 |  |
| Aspartic acid |  | (↑) | p< 0.001 |  |
| **Lysophosphatidylcholine 16 and 18** |  | (↓) | < 0.001 |  |
| **Sarcosine** | Koutros et al. (2013) | (↑) | 0.03 |  |
|  |  |  |  |  |
| **Wisteria** | De Vogel et al. (2013) | (↓) | 0.03 |  |
| Glycine/**serine** |  | (↓) | p< 0.001 |  |
| **Sarcosine** |  | (↓) | 0.03 |  |
| Betaine |  | NR | 0.21 |  |
| Dimethylglycine (DMG) |  | NR | 0.76 |  |
| **Serine** |  | NR | 0.64 |  |
| Decanoylcarnitine (C10) | Giskeodegard et al. (2015) | (↑) | 0.006 |  |
| Tetradecenoylcarnitine (C14: 1) |  | (↑) | 0.021 |  |
| Octanoylcarnitine (C8) |  | (↑) | 0.022 |  |
| Nonanoylcarnitine (C9) |  | (↑) | 0.009 |  |
| Arg |  | (↑) | 0.039 |  |
| Kinurenina |  | (↑) | 0.009 |  |
| **LysoPC a C16:0** |  | (↓) | 0.033 |  |
| **LysoPC a C18:0** |  | (↓) | 0.039 |  |
| LysoPC a C20:4 |  | (↓) | 0.025 |  |
| **PC** aa C34:4 |  | (↓) | 0.019 |  |
| **PC** aa C38:5 |  | (↓) | 0.026 |  |
| **PC aa C40:4** |  | (↓) | 0.045 |  |
| **PC** aa C40:5 |  | (↓) | 0.019 |  |
| **PC ae C38:2** |  | (↓) | 0.036 |  |
| **Valine** |  | (↑) | 0.032 |  |
| 2-Methylglutarate |  | (↑) | 0.015 |  |
| Lipid 2 |  | (↓) | 0.017 |  |
| Gln þ Glu |  | (↑) | 0.031 |  |
| Glutamate |  | (↑) | 0.049 |  |
| **Pyruvate** |  | (↑) | 0.015 |  |
| **Lysine** |  | (↑) | 0.015 |  |
| Dimethylsulfone |  | (↑) | 0.006 |  |
| Histidine |  | (↑) | 0.024 |  |
| Glucose |  | (↑) | 0.039 |  |
| Tyrosine |  | (↑) | 0.037 |  |
| **Phenylalanine** |  | (↑) | 0.011 |  |
| Cholesterol (EC) | Patel et al. (2014) | NR | < 0.05 |  |
|  |  | NR | < 0.05 |  |
| Dihydrosphingomyelin (DSM) |  | NR | < 0.05 |  |
| **Phosphatidylcholine (PC)** |  | NR | < 0.05 |  |
| Egg phosphatidylcholine (ePC) |  | NR | < 0.05 |  |
| egg Phosphatidylethanolamine (ePE) |  | NR | < 0.05 |  |
| **Phosphatidilcolina PC** 40:3/PC42:4 |  | (↑) | < 0.0001 |  |
| ePC 38:5 |  | (↓) | 0.0007 |  |
| C19:CE |  | NR | <0.0001 |  |
| C20:0CE |  | NR | <0.0001 |  |
| C20:1CE |  | NR | <0.0001 |  |
| C20:2CE |  | NR | 0.0014 |  |
| DSM 16:0 |  | NR | 0.0063 |  |
| LPE 16:0 |  | NR | 0.0037 |  |
| PC 38:0 |  | NR | 0.0050 |  |
| PC40:2 |  | NR | <0.0001 |  |
| PC40:3 |  | NR | <0.0001 |  |
| PC40:7 |  | NR | 0.0011 |  |
| PC 42:10 |  | NR | 0.0004 |  |
| PC 42:2 |  | NR | <0.0001 |  |
| PC 42:3 |  | NR | <0.0001 |  |
| PC 42:4 |  | NR | <0.0001 |  |
| PC 42:5 |  | NR | <0.0001 |  |
| PC 42:8 |  | NR | <0.0001 |  |
| PC 42:9 |  | NR | 0.0002 |  |
| ePC 36:1 |  | NR | <0.0001 |  |
| ePC 36:5 |  | NR | 0.0040 |  |
| ePC 38:1 |  | NR | <0.0001 |  |
| **ePC 38:2** |  | NR | <0.0001 |  |
| ePC 38:3 |  | NR | <0.0001 |  |
| ePC 38:5 |  | NR | 0.0007 |  |
| ePC 38:6 |  | NR | 0.0053 |  |
| ePC 40:2 |  | NR | <0.0001 |  |
| ePC 40:3 |  | NR | <0.0001 |  |
| **ePC 40:4** |  | NR | <0.0001 |  |
| ePC 40:5 |  | NR | <0.0001 |  |
| ePE 34:1 |  | NR | 0.0001 |  |
| ePE 36:3 |  | NR | 0.0072 |  |
| ePE 38:0 |  | NR | 0.0022 |  |
| LysoPE(0:0/18:2) | Zang et al. (2014) | (↓) | 0.05 |  |
| LysoPE(18:2/0:0) |  | (↓) | 0.05 |  |
| **LysoPC(18:2/0:0)** |  | (↓) | 0.05 |  |
| Nonanedioic acid (azelaic acid) |  | (↓) | 0.05 |  |
| Uric acid |  | (↑) | 0.05 |  |
| Tryptophan |  | (↑) | 0.05 |  |
| **LysoPC(18:0/0:0)** |  | (↑) | 0.05 |  |
| 13-Oxo-9,11- ácido tridecadienoico |  | (↑) | 0.05 |  |
| 3-Hydroxytetradecanedioic acid |  | (↓) | 0.05 |  |
| 6-Hydroxypentadecanedioic acid |  | (↓) | 0.05 |  |
| 5-(2-Methylpropyl)-2-oxooxolane-3- |  | (↑) | 0.05 |  |
| Carboxylic acid |  | (↓) | 0.05 |  |
| 5-Butyl-2-oxooxolane-3-carboxylic acid |  | (↓) | 0.05 |  |
| LysoPE(0:0/18:2) |  | (↓) | 0.05 |  |
| LysoPE(18:2/0:0) |  | (↓) | 0.05 |  |
| LysoPC(18:2/0:0) |  | (↓) | 0.05 |  |
| Cortolone-3-glucuronide |  | (↑) | 0.05 |  |
| Pregnanetriol glucuronide |  | (↑) | 0.05 |  |
| Androstenedione |  | (↑) | 0.05 |  |
| Decanoic acid (capric acid) |  | (↑) | 0.05 |  |
| Menthol glucuronide |  | (↓) | 0.05 |  |
| Citronellol glucuronide |  | (↓) | 0.05 |  |
| l-α-Amino-1*H*-pyrrole-1-hexanoic acid |  | (↑) | 0.05 |  |
| LysoPC(0:0/18:2)b |  | (↓) | 0.05 |  |
| **Phenylalanine** |  | (↓) | 0.05 |  |
| 3β,16α-Dihydroxyandrostenone sulfate |  | (↑) | 0.05 |  |
| 2-Tert-butyl-1,4-benzenediol sulfate |  | (↑) | 0.05 |  |
| Indoxyl-sulfuric acid |  | (↑) | 0.05 |  |
| 9,10-Dihydroxi-12Z,15Z-octadecadienoic ácido |  | (↑) | 0.05 |  |
| 12,13-Dihydroxi-9Z,15Z-octadecadienoico ácido |  | (↑) | 0.05 |  |
| 15,16-Dihydroxi-9Z,12Z-ácido octadecadienoico |  | (↓) | 0.05 |  |
| 27-nor-5β-colestano-3α,7α,12α,24,25-pentol glucuronide |  | (↑) | 0.05 |  |
| Hexadecanedioic acid |  | (↑) | 0.05 |  |
| **Glutamine** |  | (↓) | 0.05 |  |
| Heptadecanoic acid |  | (↑) | 0.05 |  |
| n-[(3α,5β,7β)-7-Hydroxy-24-oxo-3-(sulfoxy)cholan-24-yl]-glycine |  | (↓) | 0.05 |  |
| n-[(3α,5β,7α)-3-Hydroxy-24-oxo-7- (sulfoxy)cholan-24-yl]-glycine |  | (↓) | 0.05 |  |
| Glycochenodeoxychholate-3-sulfate 5-isopropyl-2-methylphenol sulfate (carvacrol sulfate) |  | (↓) | 0.05 |  |
| 5′-Carboxy-α-chromanol glucuronide |  | (↑) | 0.05 |  |
| Indole-3-carboxaldehyde |  | (↑) | 0.05 |  |
| Androsterone sulfate |  | (↑) | 0.05 |  |
| 5α-Dihydrotestosterone sulfate |  | (↓) | 0.05 |  |
| Ethiocholanolone sulfate |  | (↓) | 0.05 |  |
| Stearoyl-arachidonoyl-GPE | Albanes et al. (2017) | (↓) | 0.019 |  |
| Stearoyl-linoleoyl-GPE |  | (↓) | 0.025 |  |
| Glycolithocholate sulfate |  | NR | 0.029 |  |
| Euricoyl-sphingomyelin |  | NR | 0.022 |  |
| 5-α-pregnan-3β,20α-diol disulfate |  | NR | 0.099 |  |
| Imidazole lactate |  | (↑) | 0.033 |  |
| 3-Methylhistidine |  | (↑) | 0.012 |  |
| N-Acetyl-3-methylhistidine |  | (↑) | 0.00024 |  |
| O-Cresol sulfate |  | NR | 0.039 |  |
| N-Acetylarginine |  | NR | 0.092 |  |
| N-Acetylcitrullin |  | NR | 0.028 |  |
| β-Hydroxy isovalerate |  | NR | 0.019 |  |
| L-Urobilin |  | NR | 0.031 |  |
| 2'-Deoxyuridine |  | (↑) | 0.0046 |  |
| γ-glutamyltryptophan |  | NR | 0.027 |  |
| 3-Ethylphenylsulfate |  | NR | 0.0047 |  |
| 2-Ethylphenylsulfate |  | NR | 0.034 |  |
| **Urine** | | | |  |
| F2-Isoprostane | Barocas et al. (2011) | (↑) | 0.001 |  |
| **Sarcosine** | Cao et al. (2011) | (↑) | 0.05 |  |
| 17-Epiestriol | Kosti et al. (2011) | (↓) | 0.05 |  |
| 16-Cetoestradiol |  | (↓) | 0.03 |  |
| Hydroxybutanoic acid | Struck-Lewicka et al. (2015) | (↓) | 0.035 |  |
| Cresol |  | (↓) | 2.4 E -06 |  |
| Succinic acid |  | (↓) | 0.011 |  |
| Benzoate |  | (↓) | 0.001 |  |
| Oxoproline |  | (↓) | 0.001 |  |
| Leucine |  | (↓) | 0.005 |  |
| Indoxil |  | (↓) | 0.0001 |  |
| Phenylacetamide |  | (↓) | 0.0007 |  |
| Threonic acid |  | (↓) | 0.002 |  |
| **Glutamine** |  | (↓) | 0.028 |  |
| Ureidoisobutyrate |  | (↓) | 0.0008 |  |
| Hydroxyglutarate |  | (↓) | 6.3 E -09 |  |
| α-Oxo-benzeneacetic acid |  | (↓) | 0.029 |  |
| **xanthine** |  | (↓) | 0.0004 |  |
| Acetamidopentanoate |  | (↓) | 0.005 |  |
| Dihydroxyquinoline |  | (↓) | 0.002 |  |
| Dehydrodeoxy fuconoate |  | (↓) | 3.2 E -06 |  |
| Xylonate |  | (↓) | 0.001 |  |
| Methylxanthine |  | (↓) | 0.005 |  |
| Indole acetate |  | (↓) | 0.006 |  |
| Citrulline |  | (↓) | 8,1501 E -06 |  |
| Propylmalate hippuric acid |  | (↓) | 0.049 |  |
| Hexose |  | (↓) | 9.52 E 05 |  |
| DImethyl xanthine |  | (↓) | 0.006 |  |
| Tyrosine |  | (↓) | 2.14 E -06 |  |
| Dihydroxyphenylpropanoate |  | (↓) | 0.006 |  |
| Adrenaline |  | (↓) | 2.14 E -05 |  |
| Azelaic acid |  | (↓) | 0.002 |  |
| Kinurenate |  | (↓) | 9.39 E -05 |  |
| Dihydroxyhipuric acid |  | (↓) | 0.023 |  |
| Dimethyluric acid |  | (↓) | 0.04 |  |
| Acetyl amino - aminomethyluracil |  | (↓) | 0.0018 |  |
| Tryptophan |  | (↓) | 0.002 |  |
| Indolactate |  | (↓) | 0.0002 |  |
| Hydroxybutane tricarboxylate |  | (↓) | 1.18 E -05 |  |
| Trimethyluric acid |  | (↓) | 0.0002 |  |
| Glutamyl amniobutyraldehyde |  | (↓) | 0.007 |  |
| Propanoylcarnitine |  | (↓) | 0.005 |  |
| Pantothenic acid hydroxy-tryptophan |  | (↓) | 0.036 |  |
| Butiril carnitine |  | (↓) | 0.002 |  |
| C16 sphingosine |  | (↑) | 0.033 |  |
| Methylinosine |  | (↓) | 0.003 |  |
| Xanthosine |  | (↓) | 0.004 |  |
| Octanoylcarnitine |  | (↓) | 0.002 |  |
| Methylguanosine |  | (↓) | 0.0002 |  |
| Dimethylheptanoylcarnitine |  | (↓) | 0.003 |  |
| Dimethylguanosine |  | (↓) | 0.047 |  |
| Hydroxysphingosine |  | (↓) | 0.002 |  |
| Phytosphingosine/hydroxysphinganine |  | (↑) | 0.005 |  |
| Cyclic 3′,5-AMP |  | (↓) | 0.001 |  |
| PA (12:0) |  | (↓) | 0.05 |  |
| PG (32:1) |  | (↑) | 0.007 |  |
| PG (31:4) |  | (↓) | 0.015 |  |
| Alanine |  | (↓) | 3.156 × 10^-9^ |  |
| Acetic acid |  | (↓) | 1.016 × 10^-6^ |  |
| Arabitol |  | (↓) | 1.210 × 10^-6^ |  |
| Threonine |  | (↓) | 4.061 × 10^-6^ |  |
| Glyceryl glycoside |  | (↓) | 5.479 × 10^-6^ |  |
| Sucrose |  | (↓) | 5.559 × 10^-6^ |  |
| Propanetricarboxylic acid |  | (↓) | 5.849 × 10^-5^ |  |
| Butyric acid |  | (↓) | 8.094 × 10^-5^ |  |
| Propionic acid |  | (↓) | 1.138 × 10^-5^ |  |
| Propenoic acid |  | (↓) | 2.166 × 10^-4^ |  |
| Hydroxyphenylhydroxypropionic acid |  | (↓) | 4.922 × 10^-4^ |  |
| Threonic acid |  | (↓) | 1.161 × 10^-4^ |  |
| Arabinous |  | (↓) | 1.727 × 10^-3^ |  |
| Indole |  | (↓) | 2.376 × 10^-3^ |  |
| Hydroxyhippurate |  | (↓) | 2.388 × 10^-3^ |  |
| Meso erythritol |  | (↓) | 3.548 × 10^-3^ |  |
| Isobutyric acid |  | (↓) | 4.185 × 10^-3^ |  |
| 2-Keto-l-gluconic acid |  | (↓) | 4.192 × 10^-3^ |  |
| Talosa |  | (↓) | 5.316 × 10^-3^ |  |
| Aconitic acid |  | (↓) | 5.878 × 10^-3^ |  |
| Sorbose |  | (↓) | 6.417 × 10^-3^ |  |
| Isocytric acid |  | (↓) | 6.613 × 10^-3^ |  |
| **Serine** |  | (↓) | 7.866 × 10^-3^ |  |
| **Wisteria** |  | (↓) | 1.244 × 10^-3^ |  |
| Lactose |  | (↓) | 1.321 × 10^-2^ |  |
| Hippuric acid |  | (↓) | 3.187 × 10^-2^ |  |
| Galactaric acid |  | (↓) | 3.532 × 10^-2^ |  |
| Inositol |  | (↓) | 4.093 × 10^-2^ |  |
