# Supplementary material for: Metabolomic profile and its association with the diagnosis of prostate cancer: a systematic review
Source: J Cancer Res Clin Oncol. 2024 Dec 31;151(1):29. doi: 10.1007/s00432-024-06058-w (PMC11688254; doi:10.1007/s00432-024-06058-w)
Supplement: Supplementary file 5 — Supplementary file5 (DOCX 16 KB) [file 432_2024_6058_MOESM5_ESM.docx]

| Table 3. Consistently altered metabolites in the malignancy process | | | | | |
| --- | --- | --- | --- | --- | --- |
| ***Metabolites*** | ***Sample type*** | | | | ***HMD code*** |
|  | Serum | Fabric | Urine | Seminal fluid |  |
| Sarcosine | (↑) |  | (↓) |  | HMDB0000271 |
| Pyruvate | (↑) |  |  | (↑) | HMDB0000243 |
| Valine | (↑) |  |  | (↑) | HMDB0000883 |
| Tyrosine | (↑) |  | (↓) |  | _ |
| Wisteria | (↓) |  | (↓) | (↓) | HMDB0000123 |
| Serine | (↓) |  | (↓) |  | HMDB0000187 |
| Lysine | (↑) |  |  | (↑) | HMDB0000182 |
| LysoPC C16 : 0 | (↓) |  |  |  | _ |
| LysoPC C18 : 0/Lyso PC 18:2 | (↓)/(↑) |  |  |  | _ |
| Kinurenina | (↑) |  | (↓) |  | HMDB0000684 |
| Glutamine | (↑) |  | (↓) |  | HMDB0000641 |
| PC C40 : 4/PC C42:4 | (↓)/(↑) | (↑) |  |  | _ |
| PC C40 : 5 | (↓) | (↑) |  |  | _ |
| PC C38 : 5/PC C 36:5 | (↓) | (↑) |  |  | _ |
| Xanthine |  |  | (↓) | (↑) | HMDB0000292 |
| Phenylalanine | (↑)/(↓) |  |  |  | HMDB0250791 |
| Azelaic acid | (↓) |  | (↓) |  | HMDB0000784 |

(↑) Increased concentration of the metabolite in the sample

(↓) Decreased in the concentration of the metabolite in the sample

HMD code: Code corresponding to Human Metabolome Database
