# Supplementary material for: Metabolomic profile and its association with the diagnosis of prostate cancer: a systematic review
Source: J Cancer Res Clin Oncol. 2024 Dec 31;151(1):29. doi: 10.1007/s00432-024-06058-w (PMC11688254; doi:10.1007/s00432-024-06058-w)
Supplement: Supplementary file 6 — Supplementary file6 (PDF 33 KB) [file 432_2024_6058_MOESM6_ESM.pdf]

**Appendix 1. Search strategy**

**Medline (Ovid):**

(Exp metabolomics or metabolom*.mp or exp Mass Spectrometry or (mass spectrometry).mp or (mass spectroscopy).mp or (mass spectrum analysis).mp or exp Magnetic Resonance Spectroscopy or (magnetic resonance spectroscop*).mp or (MR spectroscop*).mp or (NMR spectroscop*).mp) AND (exp Prostatic Neoplasms or exp prostatic intraepithelial neoplasia or (prostatic malignanc*).mp or (prostatic cancer).mp) AND (randomized controlled trial.pt or controlled clinical trial.pt or randomized.ab or placebo.ab or randomly.ab or trial.ab or (clinical trial).mp or (randomi*ed controlled trial).mp or exp double-blind method or Exp cohort studies or (cohort stud*).mp or exp case-control studies or (case-control stud*).mp or exp Cross-sectional studies or (cross-sectional stud*).mp)

**Central**

(Exp metabolomics or metabolom*.mp or exp Mass Spectrometry or (mass spectrometry).mp or (mass spectroscopy).mp or (mass spectrum analysis).mp or exp Magnetic Resonance Spectroscopy or (magnetic resonance spectroscop*).mp or (MR spectroscop*).mp or (NMR spectroscop*).mp) AND (exp Prostatic Neoplasms or exp prostatic intraepithelial neoplasia or (prostatic malignanc*).mp or (prostatic cancer).mp)

**Embase:**

(('metabolomics'/exp or 'metabolom*':ti,ab or 'mass spectrometry'/exp or 'mass spectrometry':ti,ab or 'mass spectroscopy':ti,ab or 'mass spectrum analysis':ti,ab or 'nuclear magnetic resonance spectroscopy'/exp or 'magnetic resonance spectroscop*':ti,ab or 'MR spectroscop*':ti,ab or 'NMR spectroscop*':ti,ab) AND ('Prostate Tumor'/exp or 'prostatic intraepithelial neoplasia'/exp or 'prostat* malignanc*':ti,ab or 'prostat* cancer':ti,ab) AND ('randomized controlled trial'/exp or 'randomi*ed controlled trial':ti,ab or 'clinical trial'/exp or 'clinical trial':ti,ab or 'double blind procedure'/exp or 'cohort analysis'/exp or 'cohort*':ti,ab or 'case control study'/exp or 'case-control stud*':ti,ab or 'cross-sectional study'/exp or 'cross-sectional stud*':ti,ab)) AND [embase]/lim
